# Psychometric properties of Rosenberg’s self-esteem scale among adolescents: a Rasch model analysis

**DOI:** 10.3389/fpsyg.2026.1704135

**Published:** 2026-03-24

**Authors:** Atika Khalaf, Rachid Ziad, Pernilla Ny, Catharina Sjödahl Hammarlund

**Affiliations:** 1 The PRO-CARE Group, Faculty of Health Sciences, Kristianstad University, Kristianstad, Sweden; 2 Hind Bint Maktoum College of Nursing and Midwifery, Mohammed Bin Rashid University of Medicine and Health Sciences, Dubai Health, Dubai, United Arab Emirates; 3 Department of Psychology, Echahid Cheikh Larbi Tebessi University, Tebessa, Algeria; 4 Department of Health Sciences, Midwifery Research, Reproductive, Perinatal and Sexual Health, Faculty of Medicine, Lund University, Lund, Sweden; 5 Department of Health Sciences, Lund University, Lund, Sweden

**Keywords:** adolescents, psychometric properties, Rasch model analysis, Rosenberg’s self-esteem scale, Sweden

## Abstract

**Background:**

The Rosenberg Self-Esteem Scale (RSES) is one of the most widely used measures of global self-esteem. While its psychometric properties have been extensively studied using classical test theory, limited evidence exists on its performance under modern test theory approaches.

**Aim:**

To evaluate the psychometric properties of the RSES using Rasch analysis in a sample of Swedish adolescents.

**Methods:**

A total of 403 adolescents (aged 9–19 years) from six schools completed the RSES. Rasch modelling was applied to assess item fit, person-item targeting, response category functioning, reliability, and differential item functioning (DIF) across sex, age, and BMI.

**Results:**

The RSES demonstrated good item fit, with most items aligning with the Rasch model assumptions. The reliability and internal consistency indicators were strong, reflecting the sound psychometric quality of the scale. The person reliability coefficient reached a high value (0.85), with PSI = 2.35, indicating the scale’s effective ability to discriminate among individuals across different levels of self-esteem. In addition, item reliability was high (0.99), accompanied by ISI = 8.38, reflecting the precision and stability of item difficulty estimates. Cronbach’s alpha was 0.87, with SEM = 2.11, all of which confirm a high level of reliability and internal consistency among the scale items. Regarding targeting, the range of participant abilities was broader than that of item difficulties, with adequate coverage of the central portion of the self-esteem continuum, though some challenges remain in representing extreme levels of the trait. DIF analyses revealed no significant bias across sex, age, or BMI, though several items displayed variation across self-esteem levels.

**Conclusion:**

The RSES showed acceptable Rasch model fit, structural integrity, and reliability among Swedish adolescents, with no evidence of DIF by sex, age, or BMI. However, suboptimal targeting and misfitting reverse-worded items limited measurement precision, particularly at very low and very high self-esteem levels. Adding items targeting the extremes and revising problematic items could enhance the RSES’s utility for adolescent self-esteem assessment in both research and clinical contexts.

## Introduction

Self-esteem has been shown in various studies to have a strong influence on cognitive processes (Wang et al., 2023), mental health (Sarfika et al., 2025), motivation (Yang et al., 2023), emotions (Fernandes et al., 2022), and behavior (Martínez-Casanova et al., 2024). Self-esteem refers to a sense of self-worth (Rosenberg, 1965). The Rosenberg Self-Esteem Scale (RSES) is a widely used measure of self-esteem, consisting of 10 items answered on a four-point Likert-type scale. The scale has been divided into two 5-item facets: self-competence and self-liking, which allows for a more nuanced understanding of self-esteem, as it captures both confidence in one’s abilities and feelings of self-worth (Sinclair et al., 2010). The validity and relevance of the RSES for measuring self-esteem have been demonstrated in different cultures and languages (García et al., 2019). Research has demonstrated that higher self-esteem is associated with a reduced risk of mood and anxiety disorders, indicating its protective role in mental health. The RSES has been used in diverse populations, such as African American single mothers (Hatcher and Hall, 2009) and rural residents in China (Du et al., 2016), demonstrating its applicability across different demographic groups. With high self-esteem, the individual reacts with greater perseverance in the face of problems. With low self-esteem, the individual is more likely to give up more easily. Both ways act as self-fulfilling prophecies that either give rise to positive or negative self-affirming behavior patterns. Heatherton and Wyland (2003) and Baumeister et al. (2003) found extensive evidence indicating that individuals with high self-esteem tend to be healthier, happier, and more productive at work. The RSES has several advantages over other self-esteem scales, and it has been translated into 28 languages and used in 53 countries in numerous studies across various cultures and populations (Park and Park, 2019; Yamasaki, 2019). Another advantage is its simplicity; the RSES consists of 10 items answered on a four-point Likert-type scale, making it easy for respondents to understand and complete (Rosenberg, 1965). This cross-cultural validity is a significant advantage when studying self-esteem in diverse populations.

Despite its strengths and widespread use, the RSES has noted vulnerabilities, especially relevant for adolescent and translated administrations, including susceptibility to social desirability, method effects from negatively worded items, limited item spread, and questions about strict unidimensionality (Classen et al., 2007; Park and Park, 2019; Su, 2022). These concerns matter in Swedish adolescents because developmental factors (e.g., evolving reading comprehension and response styles) and linguistic nuances can alter how response categories are used and whether items function equivalently across subgroups.

One method that directly addresses these issues is Rasch analysis, by testing category functioning and threshold ordering, evaluating item fit and local dependence (which can arise from negative wording), examining targeting of item difficulty to the adolescent trait distribution, assessing measurement invariance via differential item functioning (DIF) across the main available subgroups in our sample (e.g., gender, age group, and BMI), and providing interval-level person measures suitable for research and monitoring (Andrich and Marais, 2019; Boone, 2016; Tavakol and Dennick, 2013). In addition, Rasch analysis enables the transformation of ordinal raw scores into interval-level measures on a common latent continuum. This property is particularly important for self-esteem research, as raw total scores are often treated as if they are equally spaced, despite the ordinal nature of Likert-type responses. Converting raw RSES scores into interval measures allows more accurate interpretation of score differences across the self-esteem continuum and supports valid comparisons and monitoring of change (Andrich, 1978; Tennant and Küçükdeveci, 2023). Accordingly, the present study includes an examination of the raw-to-interval score conversion to illustrate how observed scores map onto underlying self-esteem levels. All these characteristics of the Rasch analysis will be explained further under the methods section.

Although the RSES shows good psychometric performance in many contexts, compared to other self-esteem instruments (Supplementary material 1), most evaluations have used classical test theory methods, e.g., Mimura and Griffiths (2007), Robins et al. (2001), and Sinclair et al. (2010). In a large U.S. sample, Sinclair et al. (2010) reported satisfactory item–total correlations, high internal consistency (*α* ≈ 0.90), minimal floor and ceiling effects, a largely unidimensional structure with self-competence and self-liking facets, and comparable reliability and factor structure across major demographic subgroups, supporting its structural, convergent, and discriminant validity. Other studies similarly document good internal consistency, test–retest reliability, (bi)factorial structure, and construct validity for the RSES in adult and youth samples from diverse cultural settings. Nevertheless, we found no Rasch-model evaluations of the RSES in Nordic populations. These adolescent- and context-specific questions warrant a Rasch evaluation in Swedish youth. Accordingly, our aim was to evaluate the psychometric properties of the Swedish RSES in adolescents using Rasch analysis, with *a priori* checks of unidimensionality, local dependence, threshold ordering, targeting, and DIF.

### Research questions

- Does the Swedish RSES exhibit essential unidimensionality in adolescents (after accounting for potential wording effects)?
- Are the four response categories used as intended, with ordered thresholds?
- Do items show acceptable Rasch fit and minimal local item dependence?
- Is the item hierarchy well targeted to the adolescent self-esteem distribution, with adequate reliability for group-level use?
- Is measurement invariant across key subgroups (sex, age, and BMI)?

## Methods

The Rasch model was used to examine the psychometric properties of RSES among adolescents in Sweden. This analysis was chosen because it allows estimation of item location independent of person parameters, which is one of the major advantages of modern test theory (Cano and Hobart, 2011; Westergren and Hagell, 2014). In this study, “modern test theory” refers to item response theory approaches, specifically the Rasch model, which estimates person locations and item parameters on a common latent self-esteem continuum. Rasch analysis provides indices of person and item reliability and information functions that indicate the precision of measurement across the trait range, evaluates structural and construct validity through tests of unidimensionality, item fit, local dependence, response category functioning, and targeting, and formally assesses measurement invariance via differential item functioning (DIF) across relevant subgroups (sex, age group, and BMI). Together, these features offer a rigorous framework for refining the RSES and ensuring that it functions as a robust measure of self-esteem in both clinical and research contexts. Thereby, this method provides detailed information on the instrument’s reliability and validity regardless of age, sex, and Body Mass Index (BMI) (Cano and Hobart, 2011).

### Participants

A convenient sample of 403 adolescents, aged 9 to 19 years (WHO, n.d.), was recruited in the Southern region of Sweden. In total, six schools participated, and the distribution of the age groups, sex, and BMI is shown in Table 1. Our 9–15-year group thus captures late childhood through early/mid-adolescence, corresponding largely to the period of compulsory schooling in Sweden, whereas the 16–19-year group reflects late adolescence, aligned with upper secondary education and associated increases in autonomy and role transitions. The sample was not designed to be statistically representative of all Swedish youth; rather, schools were selected to provide practical access to the relevant school stages and age range needed for item-level psychometric evaluation.

**Table 1:** Characteristics of the participants (*n* = 403).

Variable	Frequency	Valid percentage (%)	BMI/mean (SD)
Age group			
9–15 years	210	52.1	10.41 (9.78)
16–19 years	193	47.9	12.38 (9.7)
Sex			
Girls	171	42.4	11.48 (9.72)
Boys	232	57.6	11.26 (9.85)

Written information was sent to all parents of adolescents younger than 15 years, and signed consent was returned via the schools’ internal electronic communication systems. A customized version of the information was sent to all adolescents. According to Swedish ethical regulations, adolescents aged 15 or older can give informed consent without parental permission. Therefore, written information was sent directly to them. Data collection was initiated with verbal informed consent.

### Instrument—RSES

The RSES was originally developed in a large sample of U.S. high-school juniors and seniors (Rosenberg, 1965; Sinclair et al., 2010). Several authors recommend its use primarily from early adolescence (around 12 years) onwards and highlight that the original scale has not been formally validated for younger children (<12 years), for whom modified child versions have been proposed, e.g., Wood et al. (2021). Our inclusion of participants aged 9–11 years, therefore, extends slightly below the conventional age range and underscores the need to scrutinize item functioning and response category use across late childhood and adolescence using Rasch analysis.

The scale measures “global self-esteem,” that is, a person’s overall evaluation of their worth and general attitude toward themselves, rather than self-esteem in specific domains (e.g., academic or social). It contains 10 items, of which five are positively worded statements (1, 3, 4, 7, 10) and five are negatively worded items (2, 5, 6, 8, 9) and thus are reverse scored. The responses are “Strongly Disagree” 1 point, “Disagree” 2 points, “Agree” 3 points, and “Strongly Agree” 4 points. The sum scores for all 10 items range from 10 to 40 points. A total score less than 15 indicates problematic low self-esteem, whilst higher scores indicate higher self-esteem.

The instrument has also shown good internal reliability (Cronbach *α* = 0.85–0.90) among a group of 12- to 19-year-old adolescents (Bagley et al., 1997) and good internal validity, with Cronbach α ranging from 0.90 (girls) to 0.84 (boys) in Sweden (Lemoine et al., 2018). The RSES is in the public domain and may be used without explicit permission; accordingly, no additional authorization was required to administer the scale in this study. We used the Swedish translation reported in Lemoine et al. (2018), who translated the RSES using a forward-backwards procedure, and confirmed a unidimensional factor structure with good internal consistency (α ≈ 0.78–0.90) across Danish, Portuguese, and Swedish boys and girls aged 12–19 years, indicating that the translated versions assess the same global self-esteem construct as the original scale. Thus, we retained all 10 items and the four-point Likert response format.

### Data collection

All six schools had reserved 1 day for all classes to complete the survey. The questionnaire was available online. The link was distributed via the schools’ local network. The participants were allowed to answer the questionnaire while in school, during a specific class hour. After information was given about how to answer the RSES questionnaire, the first page, which included an electronic consent form and demographic questions, was filled in. This procedure was supervised by two assistants for each school. Two researchers were also present to answer potential questions.

Mean completion time was 15 min. Exclusion criteria were prespecified as lack of assent/consent, age outside the target range, absence at the data collection day, or withdrawal. Of 513 invited students, 403 completed the survey (response rate = 80.4%). We did not have access to individual-level information for students who did not participate and were therefore unable to directly assess nonresponse bias. Although the response rate was high and data collection occurred in routine school settings, selection bias cannot be excluded, and the sample may not fully represent all Swedish youth (e.g., students with higher absenteeism or missing consent).

### Ethical considerations

The study was approved by the Regional Ethical Review Board in Lund, Sweden (Dnr: 2016/264), and was conducted according to the Helsinki Declaration (General Assembly of the World Medical Association, 2014).

### Data analysis

All analyses were conducted in SPSS 25.0, AMOS 24.0, and Winsteps 4.0.1. We adopted a two-step strategy in which classical test theory (CTT) methods were used to examine basic scaling assumptions and structural validity, followed by Rasch model analysis (RMA) to evaluate item-level measurement properties.

#### Classical test theory

As an initial check of theoretical/structural validity, we examined item distributions, item–total correlations, and internal consistency (Cronbach’s *α*). Values of α ≥ 0.70 were considered acceptable for group comparisons, and corrected item–total correlations ≥ 0.30 were taken to indicate adequate homogeneity of items within the scale (e.g., Nunnally and Bernstein, 1994; Tavakol and Dennick, 2013).

To explore the dimensionality of the Swedish RSES, we first applied principal components analysis (PCA) to the polychoric correlation matrix. We inspected eigenvalues (>1), scree plots, and factor loadings, considering loadings ≥0.40 as salient (e.g., Costello and Osborne, 2005). We then conducted confirmatory factor analysis (CFA) in AMOS to test a one-factor “global self-esteem” model against alternative models (e.g., two-factor models distinguishing positively and negatively worded items). Models were estimated using maximum likelihood. Model fit was evaluated with *χ*^2^/df, comparative fit index (CFI), Tucker–Lewis index (TLI), root mean square error of approximation (RMSEA), and standardized root mean square residual (SRMR). Following common guidelines, we interpreted CFI and TLI ≥0.90 as indicating acceptable fit (≥0.95 as good), RMSEA ≤ 0.08 (≤0.06 as good), and SRMR ≤ 0.08 as acceptable (e.g., Hu and Bentler, 1999; Kline, 2023). The suitability of the data for factor analysis was assessed using the Kaiser-Meyer-Olkin (KMO) measure of sampling adequacy and Bartlett’s test of sphericity. The KMO value was 0.901, indicating an excellent sampling adequacy for factor analysis. Consistent with Kaiser (1974) guideline, values ≥ 0.90 suggest that the correlations among items are compact enough to yield reliable factors. Bartlett’s test was also highly significant, *χ*^2^(45) = 1668.659, *p* < 0.001, rejecting the null hypothesis that the correlation matrix is an identity matrix and confirming that the inter-item correlations are sufficient to justify factor analysis.

The factor extraction method employed was PCA, which is relatively simple and offers ease of interpretation of the factor structure, as it tends to yield high factor loadings (Snook and Gorsuch, 1989). Finally, the Varimax rotation technique was applied, as it is the most commonly used orthogonal rotation method. Varimax rotation effectively distinguishes among factors by maximizing the sum of the variances of the squared loadings.

#### Rasch model analysis (RMA)

For the Rasch analyses, we treated the RSES as an ordered polytomous scale and applied the Rasch Rating Scale Model (Andrich, 1978). We examined the following aspects of Rasch model fit:

- *Category functioning and threshold ordering*: We inspected category frequencies, average measures, and threshold estimates to verify that response categories were used in an ordered, monotonic fashion; disordered thresholds were taken to indicate problematic category functioning and a potential need for collapsing categories (e.g., Linacre, 2002; Boone, 2016).
- *Item fit and global model fit*: Infit and outfit mean-square statistics were used to assess how well each item conformed to the Rasch model. Mean-square values between approximately 0.7 and 1.3 were interpreted as indicating acceptable fit (e.g., Bond and Fox, 2013; Boone, 2016). Items with substantial misfit were examined for content or wording issues. The Wright map (Person-Item Map) was used to display the distribution of person abilities on the left against item difficulty levels on the right, all on a common logit scale. This allows for direct comparison of the alignment between the mean person locations and the mean item locations. In the Rasch model, item difficulty values greater than 0 logits indicate relatively difficult items, with higher values signifying greater difficulty; similarly, higher values for participants reflect higher ability levels (Bond and Fox, 2013).
- *Local dependence and unidimensionality*: Residual correlations between item pairs were inspected, with correlations > 0.30 above the average taken as evidence of local dependency, often linked to similar wording or content (e.g., Andrich and Marais, 2019). Unidimensionality was further evaluated by PCA of the residuals; an eigenvalue < 3.0 for the first residual contrast was interpreted as supporting essential unidimensionality (e.g., Tennant and Küçükdeveci, 2023). While using Linacre’s eigenvalue < 3 criterion and the 5% variance rule, we adopted a sequential, integrative approach rather than treating these criteria as independent thresholds. Specifically, eigenvalues from the principal components analysis of residuals were used as an initial indicator of potential secondary dimensions, the proportion of variance explained by the first residual factor was evaluated as a complementary check, and both were interpreted in conjunction with Rasch model fit indices and related diagnostics.
- *Targeting and reliability*: Person-item maps were used to assess targeting, that is, the alignment between item difficulty and the distribution of person locations on the self-esteem continuum. Person and item separation indices and associated reliability coefficients were calculated, with separation > 2.0 and reliability ≥ 0.80 considered indicative of adequate precision in differentiating levels of the latent trait (Boone, 2016; Bond and Fox, 2013). The threshold parameters are specifically addressed in the category functioning analysis.
- *Measurement in*var*iance (DIF)*: Differential item functioning (DIF) was examined for sex, age groups, and BMI categories. DIF was evaluated using item-difficulty contrasts across groups and associated statistical tests; absolute DIF contrasts ≥ 0.5 logits, combined with statistically significant tests, were considered meaningful (e.g., Tavakol and Dennick, 2013; Andrich and Marais, 2019). Items showing substantial DIF were scrutinized for potential bias.

The CTT and Rasch criteria together provided complementary evidence regarding reliability, structural/construct validity, and measurement invariance of the Swedish RSES in this adolescent sample.

We applied the Rasch Rating Scale Model (RSM) (see Supplementary material 2) for ordered polytomous data (Andrich, 1978) using Winsteps, version 4.0.1 (Linacre, 2025). The RSM is a polytomous Rasch model within the one-parameter logistic Item Response Theory (IRT) family, assuming equal item discrimination and common rating-scale thresholds across items and was used to evaluate the internal structure and item functioning of the RSES. Moreover, the RSM allows for evaluating model fit, item difficulty, and the appropriateness of the number of response categories. It also enables the estimation of the difficulty of each category by transforming an ordinal scale, such as the Likert scale, into an interval scale, thereby determining whether the response categories function effectively (Andrich, 1978). Sex, age, and BMI were chosen for DIF analyses because they were the only demographic variables collected and are known correlates of adolescent self-esteem, with systematic differences reported across sex, developmental stage, and weight status in previous research (Creese et al., 2023; Grajek et al., 2025; Joshi et al., 2023). The cut-point of 15 years of age was selected based on developmental considerations, reflecting a meaningful transition within adolescence that marks shifts in autonomy, identity formation, and peer relationships (Sawyer et al., 2018). As for the 19 for the BMI cut-off, it was selected based on clinical considerations commonly used in the literature and corresponds to established thresholds for distinguishing weight-related categories in adolescent populations (Cole et al., 2000; Onis et al., 2007).

## Results

### Classical test theory

#### Principal component analysis

The factor loading matrix showed that most items had high loadings on two factors, exceeding 0.70, thus surpassing the criterion of explaining 20% of the variance in each item (i.e., loading ≥ 0.40). Some items exhibited moderate cross-loadings on the second factor, reaching approximately 0.30, indicating slight overlap between the two factors without compromising the clarity of the factor structure. These results suggest that the items possess strong explanatory power, supporting the factorial validity of the scale.

Using the factor loadings (sums of squared loadings after rotation) and eigenvalues, it was found that only the first and second factors had eigenvalues greater than 1.0, with the eigenvalue for the first factor being 4.789 and the percentage of explained variance 47.89%, while the eigenvalue for the second factor was 1.378 with 13.78% of the variance explained. The remaining factors had eigenvalues below 1.0, indicating that the first two factors account for the largest proportion of variance in the data (Table 2).

**Table 2:** Exploratory factor analysis of Rosenberg’s self-esteem scale.

Category	Item	Component	
		1	2
Factor 1	3	0.810	
	4	0.774	
	1	0.768	0.322
	10	0.723	
	7	0.709	0.305
Factor 2	6		0.761
	2		0.758
	9		0.741
	8		0.729
	5		0.707
Eigenvalue		4.789	1.378
% of variance		47.892	13.782
Cumulative %		47.892	61.674

The total variance explained by the first and second factors together was 61.67%. Table 2 presents the results of the PCA.

#### Confirmatory factor analysis

Although Rosenberg originally concluded that the RSES is unidimensional, subsequent studies have debated whether the scale consists of one or two factors (Gray-Little et al., 1997). Therefore, the analysis was conducted using both a one-factor model and a two-factor model. In terms of estimation method, Maximum Likelihood Estimation (MLE) was used under the assumption of multivariate normality of the data, and model goodness-of-fit was assessed using the AIC, RMSEA, TLI, and CFI indices. The RMSEA value is considered indicative of good fit when it is 0.08 or lower, while acceptable CFI and TLI values are those that approach 1 (Arbuckle, 2011; Hooper et al., 2008).

The results indicated that the two-factor model provided a better fit according to all indices. For the one-factor model, the fit indices were AIC = 357.517, RMSEA = 0.142, TLI = 0.779, CFI = 0.828, and *χ*^2^(35) = 317.392. In contrast, for the two-factor model, the indices were AIC = 112.178, RMSEA = 0.051, TLI = 0.971, CFI = 0.978, and *χ*^2^(34) = 70.178. Table 3 further shows that the goodness-of-fit of the two-factor model was good and satisfactory, supporting the superiority of this model over the one-factor model (Table 3).

**Table 3:** Model fit, confirmatory factor analysis of the Rosenberg’s self-esteem scale.

Model	*χ* ^2^	df	*P*	CFI	TLI	RMSEA	LO 90	HI 90	AIC
1. Factor	317.392	35	0.000	0.828	0.779	0.142	0.128	0.156	357.517
2. Factor	70.178	34	0.000	0.978	0.971	0.051	0.034	0.069	112.178

Df, Degrees of Freedom; CFI, Comparative Fit Index; TLI, Tucker–Lewis Index; RMSEA, Root Mean Square Error of Approximation; AIC, Akaike Information Criterion.

### Rasch model analysis

#### Assumptions

Rasch residual analysis produced a first residual component eigenvalue of 2.34, indicating support for essential unidimensionality (Linacre, 2006). Nevertheless, the variance explained by this component is 11%, exceeding the recommended threshold of 5% and suggesting the presence of potential secondary dimensions or systematic patterns (Table 4). Factor analysis results indicate two distinct factors (positive and negative items), with a clear tendency toward a primarily unidimensional structure with minor secondary effects.

**Table 4:** Psychometric indicators of unidimensionality and local independence for the Rosenberg self-esteem scale.

Indicator	Method	Criterion/Threshold	Result	Interpretation/Note
Unidimensionality	Rasch residual analysis	Eigenvalue of first residual contrast < 3 (Linacre, 2006)	2.34	Acceptable by Linacre’s criterion (<3), but 11% variance exceeds 5%, suggesting possible secondary dimensions
	Exploratory and confirmatory factor analysis	Presence of two distinct factors (positive and negative items)	Two factors identified	Overall tendency toward unidimensionality with weak secondary distinctions
Local independence	Residual correlation (Yen’s Q3)	Correlations < 0.20	Highest positive correlation 0.19	No local dependency between items; high negative correlations do not affect local independence

For local independence, Yen’s Q3 residual correlations show the highest positive correlation of 0.19, below the acceptable threshold of 0.20 (Yen, 1984; Zenisky et al., 2002), supporting the assumption of local independence. The relatively high negative correlations do not affect this assumption.

#### Reliability

Person reliability was 0.85, indicating the scale effectively discriminates between different self-esteem levels among 403 participants. The person separation index (PSI = 2.35) suggests that self-esteem scores can be divided into three distinct strata. Item reliability reached 0.99, an exceptionally high value, with an item separation index (ISI) of 8.38, indicating excellent stability and differentiation in item difficulty (Andrich, 1982).

Cronbach’s alpha was 0.87, demonstrating good internal consistency across scale items. The standard error of measurement (SEM = 2.11) highlights the precision of individual estimates on the scale.

**Figure 1:**
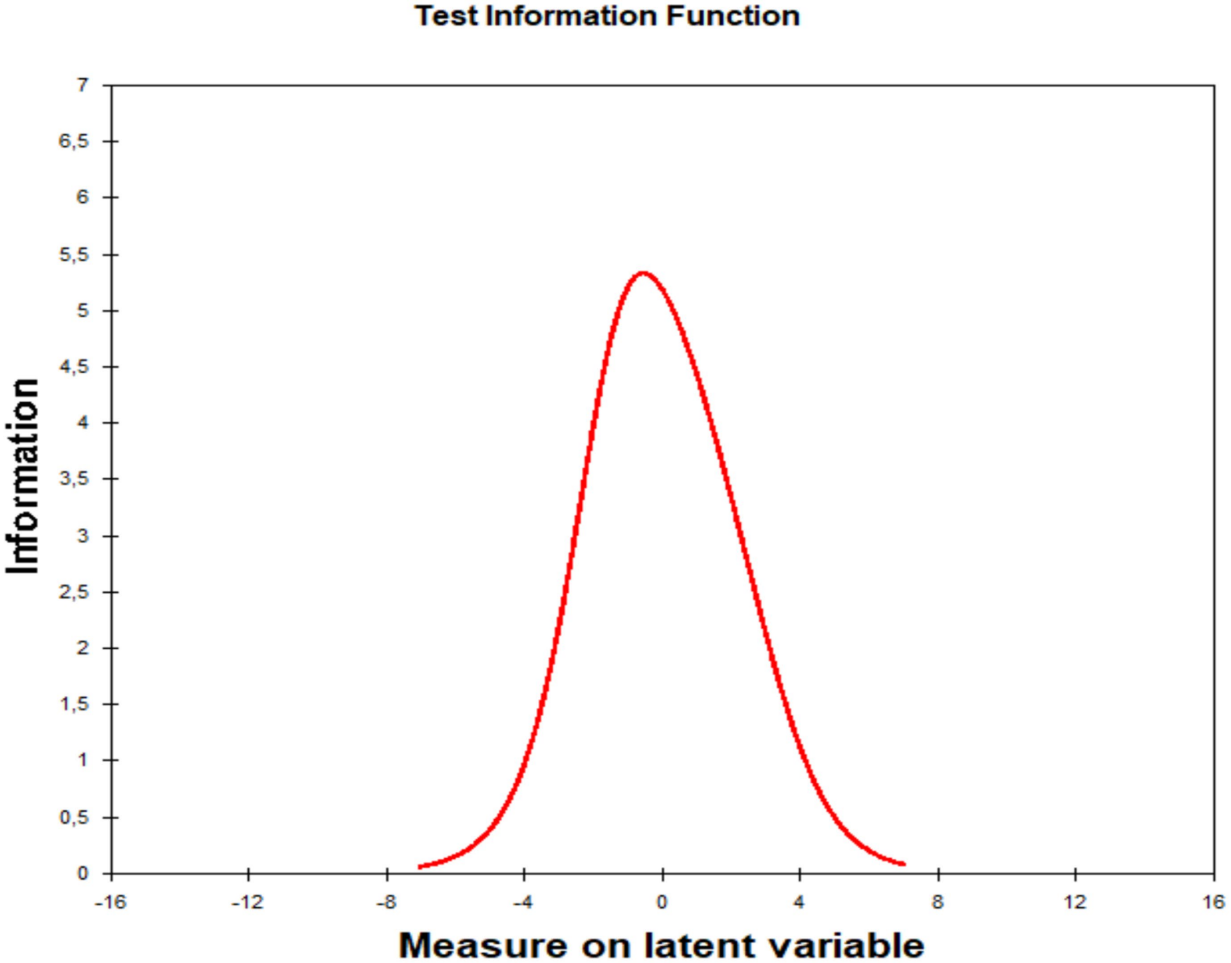
Test information function of the Rosenberg self-esteem scale.

The test information function (TIF) shows peak measurement precision near zero ability level, where information is maximized, indicating that test items are well-suited for individuals with average trait levels (Figure 1). As ability deviates toward low or high extremes, information decreases gradually, reflecting reduced test accuracy at distribution tails. The overall information curve shape suggests moderate item difficulties, highlighting the need for easier and harder items to improve precision across the full ability range.

#### Differential item functioning

Differential item functioning (DIF) analysis (Table 5) for the RSES items by gender showed that most of the 10 items showed no statistically significant differences between females and males, except for item 6. Winsteps provides DIF contrast values to estimate group differences, with values exceeding 0.64 logits indicating instability between groups (Kuang et al., 2025). DIF contrast values ranged from −0.25 to 0.10, with probabilities above the 0.05 significance level, confirming item neutrality regarding gender and absence of performance bias.

**Table 5:** Test score and differential item functioning (DIF).

Item	DIF contrast					
	Sex	Prob.	Age	Prob.	BMI	Prob.
1. On the whole, I am satisfied with myself	−0.23	0.167	−0.16	0.326	−0.13	0.587
2. At times I think I am no good at all	0.07	0.624	−0.08	0.606	0.13	0.541
3. I feel that I have a number of good qualities	−0.14	0.422	−0.08	0.657	0.17	0.490
4. I am able to do things as well as most other people	−0.13	0.459	0.08	0.655	0.04	0.860
5. I feel I do not have much to be proud of	0.00	1.000	0.00	1.000	0.02	0.916
6. I certainly feel useless at times	0.31	0.043	0.20	0.172	0.03	0.885
7. I feel that I’m a person of worth, at least on an equal plane with others	−0.25	0.144	−0.11	0.519	0.24	0.312
8. I wish I could have more respect for myself	0.05	0.714	0.00	1.000	−0.40	0.059
9. All in all, I am inclined to feel that I am a failure	0.10	0.534	0.25	0.121	−0.13	0.574
10. I take a positive attitude toward myself	0.06	0.700	−0.19	0.237	0.11	0.635

DIF contrast represents the effect size in logits or USCALE units. It is the difference between the DIF measure values for the two groups, that is, the magnitude of the difference in differential performance across the two person classifications.

DIF contrast by gender = DIF value for males − DIF value for females.

DIF contrast by age = DIF value for the age group 9–15 years − DIF value for the age group 16–19 years.

Differential item functioning contrast (DIF contrast) by body mass index (BMI) = DIF value for the first category (BMI ≤ 19) − DIF value for the second category (BMI ≥ 23). We used the quartile method to define BMI groups, selecting the first quartile (BMI ≤ 19) and the last quartile (BMI ≥ 23) to represent the two categories. This approach addressed the substantial imbalance in group sizes that arose when using a median split and ensured that each extreme BMI group contained at least 50 participants. A sample of this magnitude is typically considered sufficient for obtaining reasonably stable Rasch estimates in exploratory analyses (Linacre, 2025, p. 666). However, previous work indicates that larger samples (e.g., ≥200 participants per group) provide greater power to detect moderate DIF effects, so our DIF analyses should be interpreted as primarily screening for large DIF.

Item 6 (“I certainly feel useless at times”) was one exception, displaying statistically significant DIF with a contrast of approximately 0.31 (*p* = 0.043), suggesting it was easier for males than females. At equivalent self-esteem levels, males tended to score higher on this item, indicating a male-favorable bias that may be due to differential interpretation by gender, whereas the remaining items showed neutrality unaffected by sex differences in this sample.

DIF analysis by age group (9–15 vs. 16–19 years) revealed no statistically significant differences across all 10 items, with DIF contrasts ranging from −0.19 to 0.25 and probabilities exceeding 0.05 (Table 5). This indicates no age-related item bias, confirming equivalent self-esteem measurement across adolescent groups and supporting the scale’s validity without requiring item adjustments.

The results of the DIF contrast and the statistical significance values for the self-esteem scale items by BMI showed that all items did not exhibit statistically significant differences between the two groups (BMI ≤ 19 and BMI ≥ 23). The *p*-values ranged from 0.312 to 0.916, all of which were above the conventional significance level of 0.05. This indicates the absence of a real differential effect on item performance across BMI categories (Table 5).

However, it was observed that item 8 (“I wish I could have more respect for myself”) approached statistical significance at 0.059, with a negative DIF contrast value (−0.40), which may suggest a slight tendency for individuals with higher BMI (BMI ≥ 23) to respond differently to this item compared with those in the BMI ≤ 19 group, although this effect did not reach the conventional level of statistical significance. The remaining items showed small DIF contrast values (between −0.13 and 0.24) that were not statistically significant, which supports the validity of the scale items and their use across the two groups without bias or differences in the measurement of self-esteem (Linacre, 2025).

#### Unidimensionality

The residual analysis of the RMA indicated that the self-esteem scale accounted for approximately 49.2% of the total variance, while 50.8% remained unexplained. When examining the unexplained variance using standardized residuals analysis, the first contrast in the residuals (Unexplained Variance in 1st contrast) had an eigenvalue of 2.34, which is acceptable because it is lower than the critical value of 3 suggested by Linacre (2006) when evaluating unidimensionality. However, the proportion of variance explained by this contrast was 11%, which exceeds the recommended 5% criterion, suggesting the presence of potential secondary dimensions or systematic effects. The subsequent contrasts showed smaller eigenvalues (1.26, 1.19, 1.00, 0.95) and accounted for lower proportions of variance, ranging from 4.8 to 6.4%, indicating that these secondary dimensions were less influential.

The comparison with the factor analysis results, which indicated two distinct factors (positive and negative items), suggests the presence of two dimensions with a clear tendency toward a primarily unidimensional structure. Despite identifying two factors, the analysis of standardized residual variance in the RMA shows that the dominant pattern in the data is essentially unidimensional, as the secondary effects are relatively weak and do not substantially distort the overall structure of the scale. Accordingly, the scale can be regarded as reflecting a largely unidimensional factorial structure, with some secondary distinctions that may warrant consideration in future analyses or scale refinements.

#### Local independence

The results for the residual correlations (Yen’s Q3) showed that the highest positive correlation between items was 0.19, which is lower than the acceptable threshold of 0.20 (Yen, 1984; Zenisky et al., 2002), indicating that there is no local dependency between the items. Although some relatively high negative correlations were observed (ranging from −0.19 to −0.33) (Supplementary material 3), these do not indicate local dependency, as local dependency is typically reflected in high positive correlations. Therefore, the assumption of local independence is supported for the scale data.

#### Item fit

The results from the item fit indices table indicate that most items on the RSES fall within acceptable ranges for both Infit and Outfit statistics, suggesting that adolescents’ responses are consistent with RMA expectations. Infit MnSq values ranged from 0.71 to 1.27, while Outfit MnSq values ranged from 0.78 to 1.30; these are generally acceptable within the criterion of 0.7–1.3 established by Wright and Linacre (1994) and Linacre (2002). Item RSES-8 was an exception, showing elevated Infit (1.27) and Outfit (1.30) values accompanied by high ZSTD scores (3.7 and 4.0), which signal unexpected response variance and warrant review of its wording or function within the scale (Table 6).

**Table 6:** Item fit indices of the Rosenberg’s self-esteem scale.

Item	Measure	S.E.	Infit		Outfit		*P*-measure corr.
			MnSq	ZSTD	MnSq	ZSTD	
RSES-8	1.09	0.07	1.27	3.7	1.30	4.0	0.67
RSES-2	1.00	0.07	1.06	0.9	1.07	1.1	0.69
RSES-6	0.83	0.07	1.06	0.9	1.08	1.1	0.70
RSES-5	0.00	0.08	1.04	0.6	1.09	1.2	0.67
RSES-10	−0.19	0.08	0.93	−0.9	1.01	0.1	0.66
RSES-9	−0.24	0.08	1.05	0.7	1.13	1.6	0.67
RSES-1	−0.53	0.08	0.71	−4.3	0.78	−2.8	0.69
RSES-7	−0.58	0.08	1.02	0.3	0.95	−0.6	0.65
RSES-4	−0.60	0.08	0.96	−0.5	1.18	2.0	0.58
RSES-3	−0.78	0.07	0.83	−2.4	0.84	−1.9	0.64

MnSq, Mean Square; ZSTD, Standardized as a Z-score.

Item polarity (Point-Measure Correlation) assesses whether items function in the expected direction to confirm the hypothesized structure. Analysis results showed point-measure correlations for RSES items ranging from 0.58 to 0.70, which are relatively high and indicate strong item association with the scale’s latent total score. Values exceeding 0.30 are typically acceptable for item alignment with the overall dimension, while those above 0.50–0.60 reflect strong consistency and good performance within a unidimensional framework. The observed range (0.58–0.70) thus provides robust evidence of homogeneous item functioning and appropriate contribution to measuring the trait, supporting the unidimensionality assumption underlying the Rasch-Andrich model (Herche and Engelland, 1996). This consistency further implies a clear internal structure, with items measuring the same latent variable (self-esteem) rather than distinct subdimensions (Table 6).

#### Targeting

Person abilities ranged from approximately +5 to −2 logits, reflecting wide variability in self-esteem levels within the sample, with a greater concentration of individuals at intermediate scale levels. Item difficulties were predominantly clustered between +1 and −1 logits, corresponding to easy to moderate difficulty. Item RSES-8 appears to be the most difficult (near +1 logit), followed by RSES-2 and RSES-6, indicating that they require higher self-esteem levels to yield responses that accurately reflect true ability. In contrast, items RSES-1, RSES-3, RSES-4, and RSES-7 were the easiest, located at negative difficulty levels (around −1 logit), meaning most examinees responded to them correctly or in line with model expectations.

**Figure 2:**
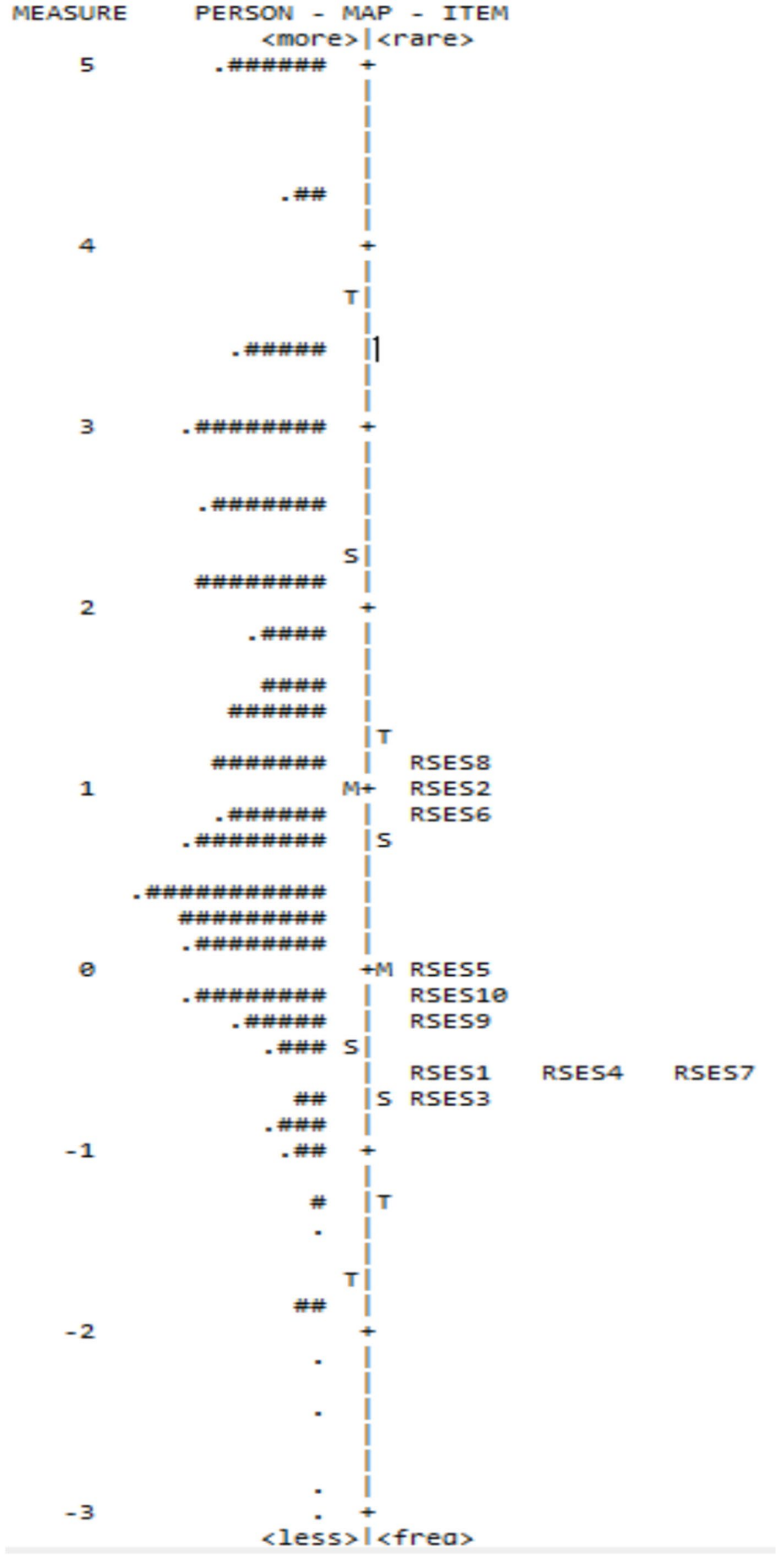
The Wright map (person-item map) displays the distribution of person abilities (on the left) against Rosenberg’s self-esteem scale item difficulty levels (on the right), on a common logit scale.

This map (Figure 2) also reveals slight mismatches between some item difficulties and person abilities. While person abilities span a broad range, items concentrate in a narrower band, suggesting that the scale may lack precision in discriminating individuals with very high or very low self-esteem. The upper and lower extremes of the ability map lack sufficient item coverage, indicating a need for additional, more challenging and easier items to enhance measurement precision across the full range of self-esteem.

The Wright map demonstrated that the scale performed well in the middle ability range but requires refinement or augmentation at the scale extremities to better represent all ability levels.

#### Ordering of response categories

The RSM assumes that as the number of response categories increases, the required ability level of the respondent rises accordingly, and that thresholds must increase progressively. Goodness-of-fit for each category should be below 2.0 on a four-point scale, while the progressive increase in Andrich threshold values should exceed 1.1 (Linacre, 2002).

The analysis of rating scale category functioning reveals that Category 1 represents the lowest level on the measured trait, recording the lowest observed average (−0.87) and usage rate (8%), with relatively elevated Outfit (1.68) and Infit (1.35) values indicating response inconsistency compared to expected patterns. Category 2 showed greater stability, with fit indices near 1, suggesting statistically expected usage. Category 3, the most frequently used (39%), demonstrated optimal measurement efficiency, with acceptable fit values (0.78) reflecting good response homogeneity (Table 7).

**Table 7:** Results of the rating scale category functioning of the Rosenberg’s self-esteem scale.

Category label	Observed count	%	Observed average	Infit MnSq	Outfit MnSq	Andrich threshold
1	307	8	−0.87	1.35	1.68	–
2	787	20	−0.24	0.84	0.88	−1.58
3	1,580	39	0.91	0.78	0.78	−0.32
4	1,356	34	2.36	1.03	1.02	1.89

Category 4, representing the highest scale level, recorded a high observed average (2.36) and acceptable response alignment despite a slightly elevated threshold (1.89), indicating that transitioning to it requires higher trait levels. Overall, the results confirm logical category ordering, with stronger performance in central categories relative to extremes (Table 7).

#### Interval measures

This section presents the conversion of ordinal raw RSES scores into interval-level Rasch measures. The grouping of raw scores into low, medium, and high ranges is used solely for illustrative purposes to facilitate interpretation of the score-to-measure relationship and does not imply the existence of discrete subgroups or heterogeneous classes within the sample. The results demonstrate a non-linear relationship between raw scores and latent self-esteem, with the greatest measurement precision in the middle of the continuum and increased uncertainty at the extremes (Table 8).

**Table 8:** Conversion table of the raw scores of the RSES-10 into interval measures of ability.

Raw score	Measure	SE
10	−5.26	1.84
11	−4.01	1.03
12	−3.26	0.75
13	−2.79	0.63
14	−2.44	0.56
15	−2.15	0.52
16	−1.89	0.49
17	−1.66	0.47
18	−1.44	0.46
19	−1.24	0.45
20	−1.04	0.44
21	−0.85	0.44
22	−0.66	0.43
23	−0.48	0.43
24	−0.29	0.43
25	−0.10	0.44
26	0.09	0.44
27	0.29	0.45
28	0.49	0.45
29	0.70	0.46
30	0.92	0.47
31	1.14	0.48
32	1.38	0.50
33	1.64	0.51
34	1.91	0.54
35	2.22	0.57
36	2.56	0.61
37	2.97	0.68
38	3.50	0.79
39	4.32	1.06
40	5.61	1.86

SE, standard error. Low category scores (10–19), Medium category scores (20–29), and High category scores (30–40).

The low category (scores 10–19) had a mean raw score of 14.5 and an interval estimate of −2.614 (SE = 0.720), indicating very low self-esteem concentrated in negative Rasch scale regions. The medium category (20–29) averaged 24.5 raw with −0.185 estimate (SE = 0.441), reflecting average self-esteem and highest precision near the center where SE is typically lowest. The high category (30–40) represented elevated self-esteem, averaging 35.0 raw with a 2.561 estimate (SE = 0.734), a common pattern at upper extremes due to increased uncertainty. This conversion provides precise self-esteem gradation, highlighting optimal estimation in the middle range.

## Discussion

To the best of our knowledge, this is the first study that employed Rasch analysis to investigate the psychometric performance of the RSES among Swedish adolescents. The findings offer valuable insights into how the scale performs under the framework of MTT, advancing beyond CTT by uncovering item-level behaviors, scale precision, and person-item targeting. Interestingly, the results from using RMA were more robust compared to the findings using CTT, in line with a previous study (Kamudoni et al., 2018). In line with previous psychometric evaluations of RSES, including in clinical and educational contexts (Park and Park, 2019; Sinclair et al., 2010), this study affirms the overall structural soundness of the scale and highlights critical areas for revision.

### Psychometric performance and model fit

The Rasch model revealed acceptable item functioning. Most items displayed satisfactory fit to model expectations with good item separation and appropriate alignment with the latent construct of global self-esteem. These findings align with recent psychometric literature emphasizing the stability and internal consistency of RSES items in adolescent and young adult populations (Čerešník et al., 2022). However, only item 8 showed signs of misfit from expected patterns. Similar concerns have been raised in recent scale validation studies using RMA, in which misfitting items were frequently associated with reverse-coded or negatively worded statements (İlhan et al., 2024; Tennant and Küçükdeveci, 2023). Comparable targeting problems have also been observed in other widely used mental-health instruments with youth or community samples; for example, a national Rasch study of the Strengths and Difficulties Questionnaire reported substantial floor and ceiling effects (Kersten et al., 2018) underscoring that mistargeting is a common challenge when scales are administered to general or adolescent populations. Collectively, these findings support revising the RSES to add harder items that capture very high self-esteem and easier items that better represent severe negative self-perception, thereby improving targeting and discrimination across the full adolescent ability range.

The Rasch residual analyses provided further support for the structural integrity of the RSES as a measure of global self-esteem. Although the first residual contrast exceeded the conservative 5% variance guideline, its eigenvalue remained below the threshold typically associated with a substantively meaningful secondary dimension. When considered alongside the pattern of subsequent contrasts, which were markedly smaller, and the overall item fit and local independence diagnostics, these findings support essential unidimensionality rather than substantive multidimensionality. This pattern is consistent with previous research suggesting that residual variance in the RSES is often attributable to minor method effects, particularly related to item wording, rather than to distinct latent constructs. Thus, the scale can be regarded as sufficiently unidimensional for measurement purposes within a Rasch framework.

### Targeting and measurement gaps

A critical contribution of RMA lies in its ability to assess the match between item difficulty and respondent ability. As mentioned earlier, the Wright map and person-item threshold distribution revealed a substantial targeting mismatch in the RSES. This discrepancy indicated ceiling and floor effects that impaired the scale’s ability to discriminate at the extremes of self-esteem (Garin, 2024). Similar patterns have been documented when the RSES is applied to other populations: item-person maps have revealed participants located above the most difficult item and measurable ceiling effects, indicating insufficiently challenging content at the top end (Park and Park, 2019).

The examination of raw-to-interval score conversion further highlighted important measurement implications. The Rasch-derived interval measures demonstrated a nonlinear relationship between observed raw RSES totals and latent self-esteem, indicating that equivalent raw-score differences do not represent equal changes across the trait continuum. Measurement precision was greatest in the middle range of self-esteem and declined toward the extremes, mirroring the targeting results observed in the Wright map. These findings underscore the limitations of relying solely on raw scores for interpretation, particularly when comparing individuals at different trait levels or monitoring change over time, and illustrate the added value of Rasch-based interval measures for more accurate interpretation (Tennant and Küçükdeveci, 2023).

### Item function and response patterns

The distribution of response categories across items further revealed potential concerns. Some items showed skewed distributions, particularly positively worded items that were too easily endorsed, limiting their utility in differentiating levels of self-esteem. This response clustering is consistent with Rasch-based evaluations of adolescent self-report instruments, showing that ceiling effects in generally healthy samples can reduce measurement precision at higher trait levels (Gong et al., 2021).

Negatively worded items also produced uneven response distributions, possibly reflecting issues with comprehension, response style, or social desirability bias. These problems are well-documented in contemporary measurement research, with studies showing that item polarity can significantly impact response accuracy and factorial structure (Zhang et al., 2016). Therefore, future versions of the RSES should consider rewording or replacing reverse-scored items to enhance scale clarity and psychometric performance.

An important contextual factor to consider is the age distribution of the sample. The majority of participants were in the 9- to 15-year-old age group (n = 210), while the 16- to 19-year-old age group accounted for a smaller proportion of the sample. Contemporary reviews indicate that identity undergoes systematic maturation (with substantial instability earlier on) throughout adolescence, which may make global self-appraisals less stable in younger adolescents and more differentiated in older adolescents (Branje, 2022). Consistent with this, longitudinal meta-analytic studies show that mean self-esteem tends to increase from mid-adolescence into adulthood, implying that older adolescents are more likely to endorse highly positive statements, contributing to the observed ceiling effect, while younger adolescents may struggle even with easy items, contributing to floor effects (Orth et al., 2018). Moreover, recent psychometric evaluations of the RSES in adolescent samples indicate that the scale’s items are often easily endorsed, and that brief, IRT-refined forms still demonstrate age-group invariance, a pattern consistent with our finding that the current item set underserves the extremes of the trait (Moksnes et al., 2024; Monteiro et al., 2022). From a measurement perspective, Rasch guidelines clarify that poor targeting results in floor and ceiling effects and weak discrimination at the ends of the continuum; accordingly, the item pool should be expanded to include harder items (e.g., stronger mastery/competence claims) and easier items (capturing severe negative self-views) to improve measurement precision across different age groups (Martin et al., 2024).

### Differential item functioning and fairness

Interestingly, the DIF analysis in this study revealed no significant bias across the demographic variables sex, age, and BMI, affirming the RSES’s fairness across these groups. However, the presence of DIF across class intervals (i.e., latent trait levels) for five items suggests that these items may behave inconsistently across self-esteem levels. This raises concerns about their ability to provide equitable measurement across the entire trait continuum. Similar response patterns have been documented in work on ideal point models for non-cognitive, socially evaluative constructs, where item responses show non-monotonic functioning across trait levels due to differential interpretations and motivational influences (Tay and Ng, 2018).

Ensuring that items function consistently across the ability spectrum is a key requirement for modern measurement tools, particularly in adolescent populations where developmental variability is pronounced. As such, future iterations of the scale should include cognitive interviews and cross-validation studies for DIF-flagged items.

### Implications and future directions

To enhance the RSES, future research should expand the item pool to capture the full range of self-esteem, especially at its lower and upper extremes. Adding new items targeting these gaps will enhance the scale’s ability to more precisely differentiate among individuals with elevated self-esteem, thereby improving its sensitivity and comprehensiveness. Additionally, these new items should be carefully formulated with consideration of cultural and social contexts to ensure their suitability across diverse target groups. Moreover, conducting longitudinal Rasch analyses would allow researchers to examine the scale’s stability and sensitivity to change over time, which is crucial for intervention studies and developmental assessments.

This study adds to the growing body of literature demonstrating the advantages of Rasch modelling in evaluating psychological instruments and highlights the continued relevance of RSES as a global tool for assessing self-esteem. Nonetheless, the scale would benefit from modern psychometric enhancements to maintain its utility in diverse populations and emerging educational and mental health contexts.

### Study limitations

The study has several limitations that should be acknowledged. The sample size and its specific demographic characteristics may limit the generalizability of the findings, particularly to populations that differ in age, socioeconomic background, or cultural context. While we do not claim that this sample is statistically representative of all Swedish youth, our sampling strategy was chosen to obtain adequate coverage of the relevant school stages (compulsory vs. upper secondary) and of the age range in which self-esteem becomes a central developmental issue (Sawyer et al., 2018). For Rasch analysis, the primary requirement is sufficient variability and coverage along the latent self-esteem continuum to calibrate items and evaluate measurement properties, rather than a probability sample designed to estimate population parameters (Linacre, 1994; Tennant and Küçükdeveci, 2023; Wright and Linacre, 1989). In addition, we did not collect individual-level data from nonresponding students and therefore could not formally compare respondents and nonrespondents on sociodemographic or psychological variables. However, the relatively high response rate (80.4%) reduces, but does not eliminate, the risk of selection bias, as nonresponse can still lead to systematic differences between respondents and nonrespondents (Groves, 2006). We cannot, therefore, rule out nonresponse bias, which limits the generalizability of prevalence or mean-level estimates of self-esteem to all Swedish youth.

For applications in other age ranges or cultural contexts, local pilot testing, cognitive interviewing of problematic items, and Rasch-based revalidation are strongly recommended before routine use. We also recommend that users complement the RSES with additional measures that better cover extreme self-esteem levels. Exercise caution when interpreting small differences or changes in total scores is also advised. Finally, we recommend considering Rasch-based or revised versions that include additional items targeting the extremes and reworded or replaced reverse-scored items.

## Conclusion

This Rasch-based evaluation of the RSES among Swedish adolescents provides important insights into its psychometric performance. Overall, the scale demonstrated good item fit and confirmed its structural integrity, with good reliability coefficients for both persons and items, reflecting stability and measurement consistency.

However, some weaknesses were identified, the most notable being poor targeting between item difficulty and participant ability, as well as some misfitting items, particularly those that were reverse-worded. These findings indicate limited sensitivity of the scale in distinguishing adolescents at the extreme ends of the self-esteem continuum, as well as a potential impact of item wording or response bias on measurement precision in some cases.

Importantly, DIF analyses revealed no significant bias by sex, age, or BMI, suggesting consistent scale performance across these demographic groups.

This Rasch-based evaluation of the RSES among Swedish adolescents demonstrates that the scale exhibits acceptable item fit, good reliability, and measurement invariance across sex, age, and BMI, supporting its use as a measure of global self-esteem in this population. At the same time, the analyses revealed suboptimal targeting and reduced precision at the lower and upper ends of the self-esteem continuum, as well as indications that reverse-worded items contribute to minor secondary effects. Importantly, the Rasch model provided a framework for expressing self-esteem scores on an interval-level scale when model assumptions were met, allowing more accurate interpretation of score differences than is possible with ordinal raw totals alone. Together, these findings affirm the continued relevance of the RSES while highlighting the need for refinement, including the addition of items targeting extreme trait levels and reconsideration of problematic item wording, to enhance its measurement precision and interpretability in adolescent research and practice.

## Data Availability

The raw data supporting the conclusions of this article will be made available by the authors, without undue reservation.
