# Supplementary material for: Psychometric properties of Rosenberg’s self-esteem scale among adolescents: a Rasch model analysis
Source: Front Psychol. 2026 Mar 24;17:1704135. doi: 10.3389/fpsyg.2026.1704135 (PMC13055173; doi:10.3389/fpsyg.2026.1704135)
Supplement: Supplementary file 1 [file Data_Sheet_1.pdf]

Supplemental Material 1

Comparison between RSES and other self-esteem instruments

A number of self-esteem instruments are available. Comprehensive reviews identify the Rosenberg Self-Esteem Scale (RSES), the Coopersmith Self-Esteem Inventory (CSEI), the State Self-Esteem Scale (SSES), the Self-Liking/Self-Competence Scale (SLCS), and the Single-Item Self-Esteem Scale (SISE) as among the most frequently used measures in the literature (Donnellan et al., 2015). The CSEI is a 50-item questionnaire that yields global and domain-specific scores (family, peers, school/work, general social activities), making it relatively lengthy and more focused on context-specific self-evaluations than on a parsimonious global self-worth construct (Potard, 2018). The SSES, in contrast, is a 20-item measure of state self-esteem, with subscales capturing performance, social, and appearance self-esteem, designed to detect short-term fluctuations rather than stable trait self-esteem (Jordan, 2018). The SLCS conceptualises self-esteem as a two-dimensional construct (self-liking and self-competence) and consists of two 10-item subscales intended to provide a more differentiated assessment of evaluative and efficacy-based aspects of self-regard (Vandromme et al., 2007). At the opposite end of the spectrum, the SISE uses a single global item as a brief proxy for self-esteem in large-scale surveys where questionnaire space is severely constrained (Looti, 2025).

The present study focuses on the Rosenberg Self-Esteem Scale (RSES), a 10-item Likert-type measure of global, trait self-esteem. The RSES is widely regarded as the most popular and extensively used instrument for global self-esteem in psychological and social science research, accounting for the largest share of citations to self-esteem measures in major journals (Donnellan et al., 2015). It has demonstrated strong internal consistency and test–retest reliability, as well as convergent and discriminant validity, across a wide range of populations (Looti, 2025; Robins et al., 2001; Rosenberg, 1965). Large cross-national studies

further support its cross-cultural applicability as a measure of global self-worth (Looti, 2025). Compared with longer, domain-specific instruments such as the CSEI or multi-dimensional state measures such as the SSES and SLCS, the RSES offers a brief, conceptually focused assessment of overall self-acceptance and self-worth that minimises respondent burden while retaining robust psychometric properties (Čerešník et al., 2022; Classen et al., 2007; Hatcher & Hall, 2009; Jordan, 2020). In addition, the scale is freely available, widely translated. These characteristics make the RSES particularly suitable for the aims of the present study, which are to model global, trait self-esteem at the item level using Rasch analysis and to allow meaningful comparison with the extensive existing literature employing the same scale (Robins et al., 2001).

These characteristics make the RSES particularly suitable for the aims of the present study, which are to model global, trait self-esteem at the item level using Rasch analysis and to allow meaningful comparison with the extensive existing literature employing the same scale.

References

Čerešník, M., Dolejš, M., Čerešníková, M., & Tomšik, R. (2022). Psychometric Analysis of Rosenberg's Self-Esteem Scale. A Specific Application of the Scale on Adolescents Aged 11-19. *TEM Journal*, *11*(4), 1732.

Classen, S., Velozo, C. A., & Mann, W. C. (2007). The Rosenberg Self-Esteem Scale as a measure of self-esteem for the noninstitutionalized elderly. *Clinical Gerontologist*, *31*(1), 77-93.

Donnellan, M. B., Trzesniewski, K. H., & Robins, R. W. (2015). Measures of self-esteem. In *Measures of personality and social psychological constructs* (pp. 131-157). Elsevier.

Hatcher, J., & Hall, L. A. (2009). Psychometric properties of the Rosenberg self-esteem scale in African American single mothers. *Issues in Mental Health Nursing*, *30*(2), 70-77.

Jordan, C. H. (2018). State self-esteem scale. In *Encyclopedia of personality and individual differences* (pp. 1-3). Springer.

Jordan, C. H. (2020). Rosenberg self-esteem scale. *Encyclopedia of personality and individual differences*, 4518-4520.

Looti, M. (2025). *Single-Item Self-Esteem Scale (SISE).* Psychological Scales & Instruments Database. Retrieved February 8 from https://db.arabpsychology.com/scales/single-item-self-esteem-scale-sise/

Potard, C. (2018). Self-esteem inventory (Coopersmith). In *Encyclopedia of personality and individual differences* (pp. 1-3). Springer.

Robins, R. W., Hendin, H. M., & Trzesniewski, K. H. (2001). Measuring global self-esteem: Construct validation of a single-item measure and the Rosenberg Self-Esteem Scale. *Personality and social psychology bulletin*, *27*(2), 151-161.

Rosenberg, M. (1965). Rosenberg self-esteem scale. *Journal of Religion and Health*.

Vandromme, H., Hermans, D., Spruyt, A., & Eelen, P. (2007). Dutch translation of the Self-Liking/Self-Competence Scale–Revised: A confirmatory factor analysis of the two-factor structure. *Personality and Individual Differences*, *42*(1), 157-167.
