# Supplementary material for: Psychometric properties of Rosenberg’s self-esteem scale among adolescents: a Rasch model analysis
Source: Front Psychol. 2026 Mar 24;17:1704135. doi: 10.3389/fpsyg.2026.1704135 (PMC13055173; doi:10.3389/fpsyg.2026.1704135)
Supplement: Supplementary file 2 [file Data_Sheet_2.pdf]

**Supplementary Material 2**

***Rasch Rating Scale Model (RSM)***

In this model, the probability that person *n* with self-esteem level $\theta_n$ endorses category *k* on item *i* is given by:

$$P(X_{ni} = k \mid \theta_n) = \frac{\exp\left(\sum_{m=0}^{k}[\theta_n - \beta_i - \tau_m]\right)}{\sum_{c=0}^{m_i} \exp\left(\sum_{m=0}^{c}[\theta_n - \beta_i - \tau_m]\right)},$$

where $\beta_i$ is the difficulty (location) of item $i$, $\tau_m$ are the step (threshold) parameters shared across items for transition between response categories, and $m_i$ is the highest category for item $i$. In this framework, item functioning is evaluated by examining whether the observed category responses align with the model-expected probabilities given $\theta_n$, $\beta_i$, and $\tau_m$. Items whose response patterns systematically deviate from the model (misfit) or show disordered thresholds (non-monotonic category structure) are interpreted as functioning poorly with respect to the latent self-esteem continuum.
