# Supplementary material for: Psychometric properties of Rosenberg’s self-esteem scale among adolescents: a Rasch model analysis
Source: Front Psychol. 2026 Mar 24;17:1704135. doi: 10.3389/fpsyg.2026.1704135 (PMC13055173; doi:10.3389/fpsyg.2026.1704135)
Supplement: Supplementary file 3 [file Data_Sheet_3.pdf]

Supplementary Material 3

The largest standardized residual correlations used to identify dependent items.

Correlation	Entry number item	Entry number item
0.19	RSES-3	RSES-4
-0.33	RSES-7	RSES-8
-0.32	RSES-3	RSES-6
-0.31	RSES-2	RSES-4
-0.30	RSES-4	RSES-8
-0.27	RSES-3	RSES-8
-0.27	RSES-9	RSES-10
-0.26	RSES-1	RSES-6
-0.26	RSES-2	RSES-3
-0.26	RSES-6	RSES-10
-0.25	RSES-1	RSES-5
-0.24	RSES-5	RSES-10
-0.24	RSES-1	RSES-9
-0.24	RSES-6	RSES-7
-0.22	RSES-4	RSES-9
-0.21	RSES-2	RSES-10
-0.21	RSES-1	RSES-2
-0.20	RSES-3	RSES-9
-0.19	RSES-2	RSES-7
-0.19	RSES-3	RSES-5
